# The quest for a unified theory on biomechanical palm risk assessment through theoretical analysis and observation

**DOI:** 10.1038/s41598-021-01679-4

**Published:** 2021-11-11

**Authors:** Peter Sterken

**Affiliations:** Unaffiliated, Plasencia, Spain

**Keywords:** Forestry, Ecological modelling, Mechanical engineering

## Abstract

Several methodologies related to the biomechanical risk assessment and the uprooting and breaking potential of palms are reviewed and evaluated in this study. Also a simple mathematical model was designed, to simulate the results of critical wind speed predictions for a tall coconut palm by using classic beam theory and Brazier buckling. First, the review presents arguments that assess the applicability of some influential claims and tree and palm risk assessment methods that have been amply marketed in the last 20 years. Then, the analysis goes beyond the classical procedures and theories that have influenced the arboricultural industry and related press so far. And afterwards, rationale behind several postulated ideas are presented, that are hoped to be fruitful in the path towards a new biomechanical theory for the biomechanical risk assessment of palms. The postulated model envisages the palm stem as a viscoelastic and hollow cylinder that is not only prone to buckling, ovalization and kinking, but also fatigue, shear, splitting and crack propagation. This envisaging was also the main reason why simple Brazier buckling formulation was experimentally applied to simulate the breaking risk of a cocostem. This study also enables a better understanding of the wide range of factors that may influence the mechanical behaviour of trees and palms under (wind) loading.

## Introduction

Palm forests and orchards are crucial to many countries’ economies. Examples are the UNESCO World Heritage Date Palm Groves in Elche, Spain and date, oil and coconut palm orchards in e.g. Africa, the Middle East, USA and the Far East. However, palms can also widely be found in urban areas which are often highly-frequented by pedestrians and vehicles. Hence, correctly assessing their structural stability is crucial. as they may cause great harm when they uproot or break. Over the last decades, commercial devices and methods have been suggested to assess the risk of palms falling down. For instance, sonic tomographs and drilling devices have been used to detect decay, or other structural defects, in palm stems^1,2^. Other methods combine a wind load analysis with breakage calculations^3,4^. And with pulling tests, trees are statically loaded with a rope to assess if they could uproot or break, comparing the pulling load with a hypothetical wind load and extrapolating measurements^3,5^. However, never has their practical applicability for palms been academically questioned in depth.

A *Nature* study showed how 70% of the researchers were unable to reproduce other researchers’ experiments and results^6^. And it also suggested that those irreproducible claims and findings could have spawned flawed research leads. Flawed leads and criteria can cause long-term societal damage, in the shape of potentially harmful commercial methods and products that, in turn, could have serious consequences regarding the physical integrity and health of its victims and ensuing legal cases^7^. Hence, and in the field of palm risk assessment and arboriculture in general, it would be not unreasonable to translate this observation into e.g. wrongly-assessed trees and palms or inappropriate evidence adduced in courts of law.

In 2018, a pine tree killed a child in the El Retiro park, Madrid, Spain, while the maximum recorded wind speed that day was only 70 km/h^8^. It was reported that the same tree had been assessed with a pulling test in 2016, and then visually assessed again a mere two days before the accident^9^. And 2 years later, a Canary date palm (*Phoenix canariensis*) fell on several people, and thereby killing a man, in Ciutadella park in Barcelona, Spain. A breeze of only 38.2 km/h had seemingly been sufficient to break the stem^10^. Risk assessment of the palm had been carried out too before the unforeseen collapse. This deadly accident triggered the implementation of risk assessments on 2026 date palms (*Phoenix dactylifera*) in Barcelona, Spain, with oscillation tests and instrumental analysis to detect inner faults in the stems^10,11^. The conclusions drawn by the City Council were based on the assessment of the collapsed palm by an external company^10^. On the webpage of that company it is claimed that they have developed a method for the risk assessment of palms with a “reliability of almost a 100%”, involving drilling and oscillation tests. The civil servant in charge also said that decayed areas in palms become problematic when 70% or more of the stem diameter is affected^12^. And that the risk of breaking would gain importance if the extent of decay exceeded at least 70% of the stem radius^13^. Nevertheless, the cavity had only affected 25% of the radius when the stem broke^13^.

Hence, these examples highlight the need to assess the scientific veracity and validity of methods, claims and criteria that have dominated the arboricultural industry and the academic field of tree and palm biomechanics in the last 20 years.

For instance: Drilling, combined with other decay detecting devices, has been explored to localise decay in palms by Ref.^14^. And also infestations and tunneling caused by Red Palm Weevil were assessed by Ref.^2^. But none of the latter quantified corresponding strength loss in the structurally damaged palm trunks. However, and on the other hand, a researcher suggested the use of a commercial microdrilling tool combined with a Visual Tree Assessment (VTA) rule, for the breaking risk assessment of coconut palms (*Cocos nucifera*)^1^. That VTA rule was supposedly a critical threshold of the ratio of the residual wall thickness versus the radius of the cross-section (*t/R*), below which hollow dicotyledon trees would be prone to breakage, which was suggested to be *t/R* = 0.32 (or 70% of the cross-section being damaged)^15^. Ever since, that easy-to-apply rule has been used world-wide for the breaking risk assessment of hollow tree and palm stems and used in combination with decay detecting tools. Also breaking safety factors of severely decayed date palm stems (*Phoenix dactylifera*) were calculated by scientists of renowned public institutions, for several wind speeds and in a highly-frequented pedestrian area^16^. A risk assessment method was also suggested that would estimate breaking safety factors for Canary date palms for a range of wind speeds^17^. And longitudinal compression stress was calculated in a Mexican Fan Palm (*Washingtonia robusta*) with a static pulling test to simulate natural wind loading^18^.

However, some of the aforementioned methods are based on simplified beam theory: a longitudinal bending stress, caused by a static bending moment, is assumed to increase linearly from the neutral axis and is then compared with a hypothetical and peripheral compression strength or Modulus of Rupture (*MOR*). Moreover, some methods marketed in the arboricultural industry seem not to adduce any robust scientific evidence to support their author's claims or theirs can seriously questioned^19,20^. And, as human lives may be at stake, one should thus wonder if the door should not be left open to scrutiny.

For instance, the abovementioned claims could briefly be contrasted with the following: under loading in the linear elastic regime, it was shown that a non-linear bending stress distribution in the palm stem had to be accounted for as bending stress, density and *MOR* would increase exponentially, and not linearly, from the neutral axis^21^. It was also asserted that the peripheral tissues are denser and stiffer, due to a higher quantity of vascular bundles, and can often be seen to be distributed as an outer ring, being this external layer that lends the trunk its main load-bearing capacity^22^. And the inner core would contain more soft tissue than vascular bundles, working as a foam-like structure with limited load-carrying abilities^22^. Nonuniformities in the mechanical properties of palm stems that set them apart from coniferous and dicotyledonous trees, such as e.g. a greater density at the stem periphery, were accounted for while the palm stem was modeled with a hollow core^23^.

Moreover, wind and *MOR* are not the only factors to take into account: for self-loading and the elastic stability (i.e. buckling) of plant stems, solutions were proposed by Ref.^24^ and e.g.^25^ in which the Modulus Of Elasticity (*MOE*) is a determining factor. Greenhill's proposal was later assessed by comparing it with other predictive formulations and real buckling of saplings and pine trees^26,27^. The assessment of the elastic stability of palm stems under their own weight (buckling) based on *MOE*, together with a wind load estimation to optimise artificial support systems, was suggested too^28^. Nevertheless, the latter could not offer any empirical evidence to support his suggestion and acknowledged that his non-profit proposal was still a hypothesis^28^. Stiffness is a determining factor for the Brazier buckling of circular hollow sections^29–31^. And the structure and *MOE* of palm stems were taken as a starting point to design conical shells for wind turbine towers subject to buckling^22^.

Hence, this brief warm-up shows already that two crucial components of palm biomechanics (*MOE* and non-linear stress and strain) seem to be neglected by some influential claims and related commercial methods and tools. This situation will be analysed more in depth further in this paper.

Next, a simplistic mathematical model was used here to support several analyses, and originated from the following: The most advanced study on palms and critical wind speeds for failure found, employed the engineering approach to calculate hypothetical static wind loads, stem displacements and hypothetical stem fibre failure for a theoretical 25 m-tall coconut palm (*Cocos nucifera*) with a 3D Finite Element software (3DFE), which did not consider dynamic loading, damping, looping or inertia effects^32^. On the other hand, a static wind load analysis model was presented, based on wind turbines, which was validated by comparing its results with those of renown softwares for tree and palm risk assessments^20^. Therein, very good agreement was obtained for all 220 simulations, while the bending stress was assumed to increase linearly from the neutral axis and stems were treated as non-deformable beams made of an isotropic material (*i.e.* the same mechanical properties in all anatomical directions, such as found in e.g. steel, rubber or butter). Nevertheless, the widely-implemented commercial software packages simulated in Ref.^20^, the results of Ref.^32^ and the methods that have been applied commercially for palm risk assessment by e.g.^1,16^, seem not to have been validated experimentally with large numbers of palms yet. And never has their practical applicability for palms been academically questioned in depth.

Hence, the following is the *first* contribution of this paper to the field of palm biomechanics and risk assessment: Several methodologies that have been suggested or marketed for the uprooting and breaking potential of palms are reviewed and evaluated. To this end, the simple model of Ref.^20^ was slightly adjusted with simple Brazier buckling formulation and a wind speed-dependent drag factor to simulate bending moments and Failure Index (*FI*) of Ref.^32^. The palm was envisaged as a hollow wind turbine tower enduring pure longitudinal bending stress and also Brazier buckling. This envisaging of the palm stem cross section as if it were a concentric ring was based on the suggestions of Refs.^21–23^. The model was intentionally maintained simplistic in structure, as it was devised to support the herein offered analysis of the scientific veracity of some influential claims and commercial methods that have been amply marketed, or used as evidence in court cases, in the last 20 years. Simple mathematical formulations and simulations can allow hidden shortcomings to see the light of day, that otherwise would not have been revealed had the study been based solely on a literature review (an example of this approach is Ref.^20^).

The *second* contribution of this paper is: exploratory analyses were conducted after observing the outcomes of the model and an exhaustive review of publications related to palm biomechanics and mechanical theories that go beyond simple beam theory. Hence, through review and theoretical analysis, several ideas are postulated that are hoped to be fruitful in the path towards a new biomechanical theory for the risk assessment of palms and trees.

## Results

The simplistic model satisfactorily simulated bending moments (*M*) and *FI* of Ref.^32^.

A wind speed-specific dragfactor (*Cd*) was found that ranged from 0.61 (at 10 m/s) to 0.19 (at 60 m/s), which enabled it to simulate the bending moments corresponding to the wind speeds. A *Cd* of 0.4 was reached at *u* = 28 m/s (this result is given here too, as several of the herein investigated methods only employ a fixed *Cd* for a fixed wind speed).

The varying theoretical *t/R* of the hollow wind turbine tower ranged from 2.248 (at *u* = 10 m/s), over 0.503 (at *u* = 23 m/s) to 0.213 (at *u* = 60 m/s) and those were the necessary parameters to simulate *FI.*

All results agreed reasonably for *M* and *FI* (see Table 1). Pearson correlation coefficient for *M* was: R = 0.9998, and for *FI* it was: R = 0.9997. Maximum difference found was 1.08% for *M* and 3.69% for *FI*. The average difference for *M* was -0.087% and 0.290% for *FI*.

**Table 1:** Comparison of moments and Failure Index between^32^ and the wind turbine model.

Windspeed (m/s)	Gonzalez (2015)^32^	Windturbine	Difference (%)
	Moment (kNm)	Moment (kNm)	Bendingmoment
10	15.9360	15.9375	0.0094
20	53.0090	52.7194	− 0.5493
23	64.4670	64.3835	− 0.1297
30	88.6270	89.4902	0.9646
40	121.1350	120.2880	− 0.7041
50	151.2170	149.3047	− 1.2808
60	178.6600	180.6093	1.0793
		Pearon Correlation Coefficient = 0.9998	Average difference = − 0.0872
			Maximum difference = 1.0793

Wind	Gonzalez (2015)^32^	Windturbine	Difference (%)
m/s	FI	FI	FI
10	0.0780	0.0776	− 0.5155
20	0.7490	0.7777	3.6904
23	1.0550	1.0421	− 1.2379
30	1.7920	1.8226	1.6789
40	2.8840	2.9391	1.8747
50	3.9780	3.9430	− 0.8876
60	5.0690	4.9419	− 2.5719
		Pearon Correlation Coefficient = 0.9997	Average difference = 0.2902
			Maximum difference = 3.6904

Lowest *FI* along the stem and for all wind speeds was found at a height of 13.1 m up the stem, whereas Ref.^32^ predicted failure between 6 and 10 m.

The alternative calculation by means of Brazier buckling, with a constant *t/R*, gave the same results too for all wind speeds, taking *MOE* into account, instead of *MOR*. However, results for Brazier buckling did not agree anymore when the height of the assessment varied, as the cocostem had been modeled as concavely tapered. However, the Brazier calculations later agreed with the breaking safety predictions according to simple beam theory (*BS*), for all heights along the stem and all wind speeds, when the stem was modeled as untapered (i.e. resembling a straight date palm trunk), with a stem base diameter of 31.647 cm and a constant shell thickness of 2.471215 cm. Pearson Correlation Coefficient was 1 and maximum difference found was 0.03% and average difference found was 0.0092%. And when the height of the assessment along the stem was maintained, *BS* and Brazier showed perfect agreement for all wind speeds (difference = 0%). However, the results for a straight stem did then, logically, not agree with Ref.^32^ anymore as the latter had modeled a concavely-tapered coconut stem (a stem base diameter of 31.647 cm and 20.9069 cm at a height of 13.1 m).

These results will be interpreted in the “Discussion” section, while arguments will also be presented that question the applicability and scientific veracity of some claims. And afterwards, rationale will be presented to postulate ideas that may serve to show the way towards a future unified theory on palm risk assessment.

## Discussion

Results agreed closely with Ref.^32^ when wind speed-specific drag coefficients were introduced that seemed to be reasonable, as the latter lie slightly under the values published by Ref.^17^ for a stiffer Canary date palm (*Phoenix canariensis*). Notwithstanding, the model's outcome on its own would not be a valid contribution to the field of palm biomechanics and risk assessment. Hence, and from here onwards, the following observations are deemed to be crucial:

The researcher^32^ used data on the mechanical properties of green tissue of senile 80–100-year-old coconut palm stems harvested in 2010 in Fiji and Samoa and which were then published in Ref.^34^. His model predicted that the critical wind speed for failure of the stem fibres was 82.8 km/h (23 m/s). However, for instance, the cyclone “Val” hit Samoa with wind speeds up to 140 knots (259.28 km/h) in 1991^35^. The real palms from Ref.^34^ that served for Ref.^32^ must have withstood, along their life span of 80–100 years, many times wind speeds that exceeded the theoretical “critical wind speed” of 82.8 km/h as predicted by Ref.^32^. And yet, those palms still stood upright when they were harvested. And no mechanically-damaged tissue in the harvested cocostems was reported in Refs.[32, 34], which suggests that those coconut palms had not suffered any failure of their stem fibres, not even when wind speeds up to 259.28 km/h hit the island they were growing on. This observation adds up to others (e.g.^10,19,20,33^) that suggest that the engineering approach as used by Ref.^32^ and used, albeit much simpler, in e.g. the “tree-statics” of Ref.^3^ may have a limited predictive value.

Next: Lowest *FI* was calculated here at a height of 13.1 m up the stem, whereas^32^ predicted failure between 6 and 10 m at 23 m/s (while the failure area would move towards the stem base with higher wind speeds). The reason behind this disagreement is simple: the herein employed model uses simple beam theory, which assumes the deformation or curvature of the stem is null. And^32^, on the other hand, depicted the bending over of the slender palm with a highly-pronounced curvature in the lower half of the stem, which would, hence, cause greater stresses. And as this happens, the upper half of the stem would align itself more with the wind and thus be less deformed, while also the experienced stresses would be lower there (see the figure in Ref.^32^ p. 126).

Furthermore, the present model “fabricated” the rising stresses, by modeling the stem as a hollow wind turbine tower with a changing *t/R* related to wind speed. The rationale behind this approach is the following: the beam theory neglects that, in palm stems, stresses increase exponentially from the neutral axis and that non-linear deformations and strong curvatures can be experienced by slender palms in high winds, as reported by e.g.^23,32^. This is thus one of the arguments against the simple beam theory in slender trees and palms, and which has been used in the present model and by e.g.^1,3–5,16–18^. One of the premises of that theory is that deformation of the beam should be small. And if, for instance, a slender Mexican Fan Palm bends over, the curvature of the stem could be too pronounced to be faithfully modeled with that theory. And real stresses due to that curvature would hence be higher than predicted. For instance, a company markets commercial pulling test on palms and shows it application on a 22-m-tall and leaning Mexican Fan Palms (La Aduana, Málaga)^36^. And theirs may thus be an untenable and risky suggestion. A crucial observation can be made here: if regular beam theory (as used in commercial pulling tests and wind load analysis software packages) had been blindly used for the coconut palm simulation (i.e*.* assuming a stiff and solid beam with no sharp rise in stresses due to the pronounced curvature of the stem and the bending stress increasing linearly from the neutral axis as if the cross-section were both full and isotropic), breaking safety factors would have been greatly *overestimated* (nearly doubled at 60 m/s) as stresses would have been *underestimated*. Which could lead to dangerous, and deadly, situations in real-life palm risk assessments.

Next: As mentioned in Ref.^20^ the results of Ref.^32^ were not validated experimentally. Hence, and even barring structural defects such as cracks or decay, it is a theoretical model that may not allow for accurate safety predictions yet. Furthermore, palm stems were reported to break either at mid-stem or just below the crown^37^. Whereas^32^ predicted failure well below mid-stem, extending towards the bottom of the stem at higher wind speeds and so, his results do not agree with real failures as reported by Ref.^37^. Also, the Red Palm Weevil (*Rhynchophorus ferrugineus*) has been said to affect the structural stability of palm trunks by excavating tunnels which could lead to their collapse^38^. The loss of structural integrity due to this tunneling is a type of defect that is not taken into account in Ref.^32^ either. And Ref.^39^ suggested several factors that may influence breakage of coconut palms subject to cyclones, and not all are taken into account in the simulation: e.g. the ratio between diameter of the bole and stem height, different mechanical failure modes related to certain hybrids (e.g. the of Malayan Red Dwarf versus Tall Palms), biomechanical degradation due to *Phytophthora palmivora* and crown characteristics (weight and volume of fronds and crop). The latter also reported fracture of the bole at the root-soil plate level and below ground, while other palms were left leaning after partial uprooting and others had their stems broken at different heights^39^. This suggests that the predictive value of the approach used by Ref.^32^ is rather limited and that, even though his is an impressive and highly-valuable contribution, it may not be extrapolable to real-life palm risk assessments yet.

Next, the commercial pulling tests and wind load analysis software of Refs.^4,5^ have been used on palms in Spain. Nevertheless, no clear references pointing towards scientific, peer-reviewed papers of theirs could be found in those publications, relating to scientifically sound data and scientifically contrasted procedures that would support their methods. Which, surprisingly, stands in sharp contrast with their criticisms toward a competitor in business: that the latter would offer (similar) methods without adducing supporting scientific evidence^4,5^. Also an influential publication asserted with a “generalised tipping curve” that the critical threshold of tilt angle for uprooting would be 2.5° (assertedly based on 400 trees), which seems to be the foundation of their commercial pulling tests^3^. That strong claim was later questioned as it still seemed unproven and thus hypothetical^19^. Moreover, the asserted results of^3^ stand in sharp contrast with those of recent researchers who, after two million measurements on more than 8000 trees, do not assert to have found any critical threshold of tilt angle yet^40^. Pulling tests have been seriously questioned of late and it is not clear to what extent their predictive value in dicotyledonous trees is reliable or not^19,20^. Which makes their use for monocotyledonous palms even more questionable as the latter are biomechanically very different from trees. Pulling tests were developed from pulling over dicotyledonous trees, with their corresponding characteristics (regarding material and geometry) of their root system and stem base. However, and on the other hand, the fleshy palm roots tolerate significant bending and twisting before they undergo mechanical failure^41^. And the roots also sprout from the stembase in a way that is similar to onions. Moreover, the effects of organic exudates from the roots on the aggregation of soil particles, which would thereby create a cement-like soil consistency, was reported too^41^. And these exudates have seemingly not been reported for dicots yet. And, thus, recommending pulling tests for palms seems to stand for blindly extrapolating hypothetical (as seemingly yet unproven) values for dicotyledons, into the unknown and unexplored realm of palms, and this is could thus be deemed very questionable.

**Figure 1:**
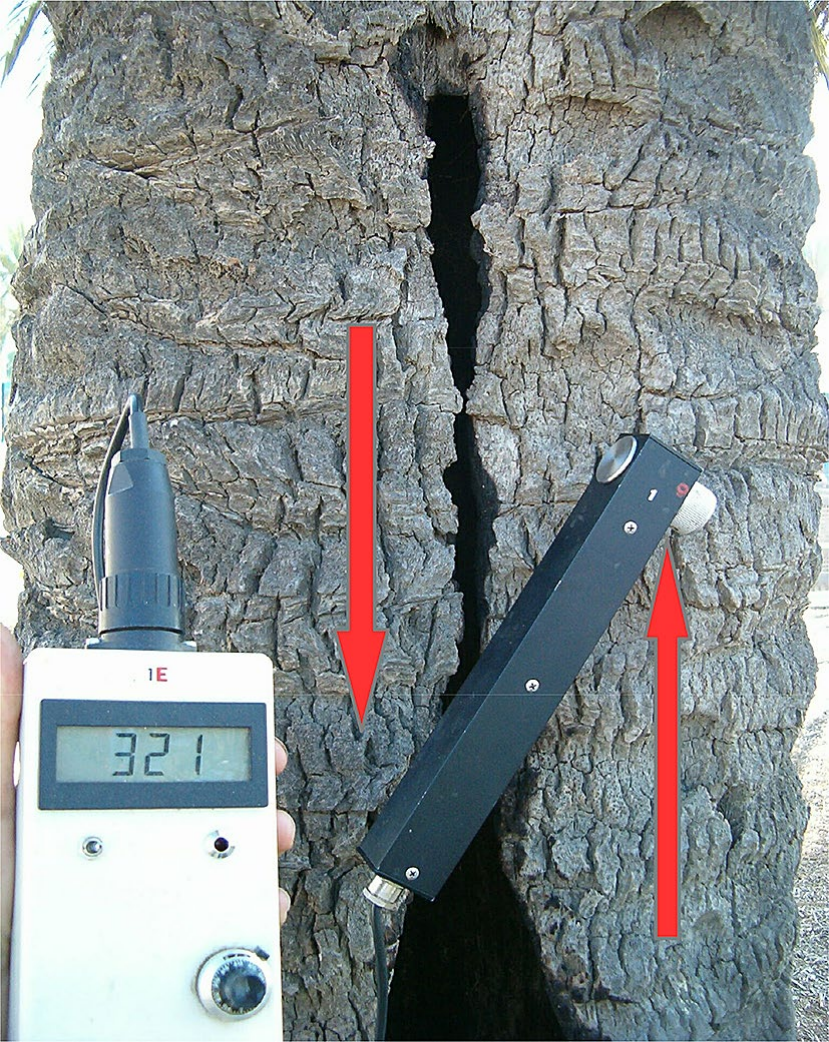
The strain gauge sensor was placed obliquely over an open cavity in a date palm, recording high shear deformations. The red arrows show the direction of the strain, producing shear as the two halves of the stem seemed to slide over each other, while a static pull was being applied.

Next, destructive experiments were experimentally carried out with the pulling test method of Refs.^3,45^ on a hollow date palm (Paseo Marítimo, Mataró, Spain) that presented a thin residual wall and a large longitudinal opening^42^. The palm was pulled with a fixed maximum load of 1.5 kN while performing 20 consecutive measurements with a strain gauge sensor or “elastometer”. The measuring tools (elastometer, inclinometer) had been provided by Refs.^3,45^. The distance between the two pins of the strain gauge sensor was 2000 mm. The direction of the pull was perpendicular to the opening of the cavity. The first 18 measurements were carried out by placing the sensor aligned with the stem (longitudinally) and in the direction of the pull, conform to the classic pulling test procedure. The highest longitudinal deformation (aligned with the stem) recorded was 0.089 mm (at 2 m height) and 0.045 mm at the height of the cavity (1 m) with a static pulling load of 1.5 kN and conform to the classic pulling test procedure of Ref.^3^. However, two alternative measurements were made afterwards by placing the sensor first in an angle of 90° (horizontally and bridging the cavity opening) and then in an angle of approximately 30° over the open cavity (Fig. 1), to assess if there could be any shear. Then, the strain gauge sensor recorded the astonishing value of 0.321 mm at the same pulling load (1.5 kN) when placed obliquely (30°) over the open cavity. Which is *more than seven-fold* the maximum axial strain measured at the same height of the stem and aligned with the pull, which indicates that shear (and not longitudinal strain) was the highest. The two sides of the open cavity seemed to *slide over each other*. This phenomenon can be visualised by bending a softcover book: the pages slide over eachother. Palm wood is much weaker regarding shear stresses and when this is coupled with extraordinary shear deformation (due e.g. a large, open cavity such as here) failure of the hollow stem can be triggered at lower loads than predicted by the beam theory. And if this extremely hollow stem exhibited high shear deformations under a transverse pull, then logic says that also other structural behaviours (e.g. ovalization, cracking or kinking) could set in.

To continue: the death of a man crushed by a Canary date palm in 2020 in Barcelona (Spain), triggered the implementation of drilling with the Resistograph of Ref.^1^ and “oscillation tests” on 2026 date palms with a “reliability of almost a 100%”, carried out by Refs.^36,43^. Nevertheless, it was stated that drilling cannot predict the residual strength of a structurally damaged trunk^44,45^. And it was shown that boring into decayed zones would probably augment the speed at which decay spreads^46^. Micro-drilling would allow fungi to grow out radially due to the microenvironment the narrow channel creates^47^. And drilling was also questioned by Ref.^48^. And correct assessments would rely on comparing results with both known standards and decay-free cores taken from the same tree, which would make this method thus highly-invasive^49^. Hence, (micro)drilling is currently highly questionable. And it was reported that the “oscillation tests” of that company were as follows: the palm is pulled manually with a rope and if the whole stem moves the palm stem is regarded as sound^50^. This was the only description found of those “oscillation tests” on palms after exhaustive online literature review. That company recommends that their oscillation test be used in all palm risk assessments^36^. Nevertheless, no clear references can be found in Refs.^36,43^ that would point to peer-reviewed publications in scientific journals that would validate their claim, so it is not clear if it is scientifically tenable or not. However, their claim could be interesting, if robust and supporting scientific evidence could be adduced and if their procedure were unbiased, documentable and reproducible by a third party.

Next, the famous “70%” criterion was adduced by the Municipal Manager of the City Council of Barcelona after the deadly accident with a Canary date palm and the manager was reported to have said that inner decay was considered problematic if at least 70% of the stem diameter was affected by it^12^. And that the risk of breaking would gain importance if the extent of decay occupied at least 70% of the stem radius^13^. However, if the palm collapsed with only 25% of its radius affected by decay, should one then not immediately refute the “70%” criterion? Notwithstanding, the applicability of this criterion as suggested for palms by Ref.^1^, can easily be refuted scientifically too: Firstly, suspicions of falsification were published regarding that highly-influential Visual Tree Assessment (VTA) rule t/R = 0.32 or 70%^51^. That t/R rule for risk assessments defined the (supposedly) allowed degree of hollowness of a tree trunk and is still being used world-wide, although it is allegedly the result of falsification^51^. Secondly, that famous “70% criterion” was, moreover, developed for dicotyledonous trees and not for monocotyledonous palms. Thirdly, tangential *MOR* (tension perpendicular to grain) in coconut wood was found to be as low as 0.233 kN/cm^2^ whereas longitudinal *MOR* could reach 5.22 kN/cm^2^^52^. And shear strength of date palm wood could be as low as 7.14% compared with its longitudinal *MOR*^52^. Even longitudinal *MOR* varied greatly between coconut, oil and date palm^52^. For oil palm (*Elaeis guineensis*), the proportion of tensile strength perpendicular to grain to longitudinal *MOR* was reported to be only 2.08%^53^. And another researcher found that tangential *MOR* would be 36.77% approximately of longitudinal *MOR* in coconut palms^32^. Therefore, tangential cracking followed by longitudinal splitting in straight hollow stems would thus be triggered earlier in date and oil palms than in coconut palms. Palm wood is highly anisotropic compared to dicotyledonous hardwood trees and, above all, there are great differences both among different palm species and even within the same palm species, depending on e.g. age and growth. And, hence, a fixed *t/R* rule seems to be an untenable recommendation for palms. And this would even not be applicable in stems with side openings or those with only heart decay^30^. Or with irregularly-distributed pockets of decay combined with cracks or invaginations. Even if one dared to leave aside the fact that high stresses can be caused by strong curvatures of slender palms in high winds (see e.g.^23,32^) and those are not taken into account in the VTA *t/R* rule of 70%. Cross-sectional flattening or ovalization, leading to cracking of thin-walled hollow stems, is neglected by classic beam theory too, while those structural failures depend highly on the *MOE* of the wood^54^. And *MOE*, *MOR* and density vary greatly according to species, age, *et cetera* and thus preclude a fixed t/R rule entirely from being useful when the aim is to predict the structural collapse of (especially) palms.

Next: Scientists published breaking safety factors and critical wind speeds for severely decayed date palms in a highly-frequented area in a major Spanish city^16^. Their suggestion that those decayed palms would withstand wind speeds of at least 135 km/h even made headlines^55^. They did not fully explain the underlying methodology in their online-published report. However, the manual of the acoustic tomograph (Fakopp^56^) they used, suggests that breaking safety factors and critical wind speeds were calculated by means of simple beam theory, which is based on longitudinal stress and strength, and assumes that wood is a homogeneous material (i.e. isotropy). However, palm wood is highly anisotropic, meaning that shear, delamination, torsional, radial and tangential tensile stresses could cause structural collapse, especially in decayed palm stems, *even if* the beam theory were applicable. So, their claim that decayed palm stems in a market square would withstand hurricane-type winds, is possibly based on what looks like a methodological error on their behalf. On the other hand, a method for the breaking risk assessment of Canary date palms was offered in a scientific paper through beam theory and a hypothetical static wind load^17^. But, fortunately, the latter acknowledged that their approach could not be valid for decayed stem areas due to shear and that failure due to progressive fatigue was not considered either. And a highly-cited researcher calculated longitudinal stress in a Mexican fan palm (*Washingtonia robusta*), seemingly based on the assumption that bending stress would increase linearly from the neutral axis and by means of a static pulling test combined with simple beam theory^18^. However, the herein offered remarks suggests that the latter's approach for palms may be a simplification that has room for improvement too. A Spanish researcher published results from static *bending* tests of planks, sawn from a Canary date palm, to be used in the context of mechanistic risk assessment models^57^. Unfortunately, the procedure he used precludes his results from being useful in that context, as current models need *MOR* and *MOE* values obtained from *compressive* axial and tangential *tensile* tests. Nevertheless, in his review he rightly acknowledged the dubious efficiency of published risk assessment models, as they would depend too much on a wide palette of unknown variables (e.g. *Cd*, *MOE*, *MOR,* density) and that decay detecting tools were not efficient, as reference values did not exist (e.g. to calculate strength loss in comparison with sound palm wood)^57^.

Next: Some readers will surely feel tempted to use the model for, and extrapolate the results to, commercial palm risk assessments. But that would clearly be premature, as can be inferred from the following observations: a wind-speed-specific drag coefficient was proposed herein to simulate the coconut palm of Ref.^32^. But a sturdy stem (in contrast with the studied 25-m-tall and flexible stem) would need to be modeled with a different *Cd*. And to make *Cd* transferrable to other palms, non-linear deformation would have to be taken into account. This non-linearity is the result of the slenderness, anisotropy and geometry of the stem, the flexibility of the crown and overall out-of-phase damping. A greater curvature of the stem leads to higher three-dimensional stresses (longitudinal, radial and tangential). And Poisson's ratios also determine deformation of the fibres^32^ and this may differ too. And all of this clearly exceeds the capacities and predictive power of simple beam theory. Also, wind drag has commonly been estimated as being proportional to the square of the wind speed. However, it was shown that this estimation may be too high for flexible culms, as at higher wind speeds the drag would be linearly proportional^58^. And, hence, real loads would be lower than predicted. They also found that the risk of mechanical damage was comparatively lower at higher wind speeds, as the plants' height was reduced by up to 45%^58^. It was suggested that coconut palms would resist hurricanes better than dicotyledonous trees because of the same strategy^32^. On the other hand, common sense and observation suggest that the mass of palms (stem, crown and crop) combined with violent gusts may lead to dynamic loading that far exceeds predictions that take into account static loading only. Two simulation studies did not consider dynamic loading, damping, looping or inertia effects^20,32^. Nevertheless, the results the first agreed very well with commercial softwares that, assertedly, would include dynamics and natural frequencies^20^. For instance, it was asserted that “and statics integrated methods that combine static pulling with dynamic wind load assessment (Wessolly 1991; Brudi and van Wassenaer 2002; Detter and Rust 2013)” (sic)^59^. Related authors also suggested that a natural frequency factor was incorporated in their calculation of the wind load and bending/uprooting moment of their pulling tests^3^. However, robust scientific evidence that would support their claim was not found and neither did the mathematical simulations find any evidence of dynamics^20^. Not including the influence of dynamics (e.g. the swinging of slender trees and palms) in a wind load analysis could underestimate real wind-induced loads. Which means that the palm or tree could thus fall down even if it had been assessed as “safe”. Also the weight of crop (e.g. dates or coconuts) could add inertial forces to the swinging and this could be a subject for future research on wind loads in palms^20^.

Further: Mechanical properties (strength, stiffness and density) of green palm tissue are still a relatively unexplored field, although several palm species have been studied^17,22,32,52,53,60–62^. Properties from these publications of other palm species could be introduced in the model to explore their importance relative to other influencing factors such as slenderness, wind speeds, loads, *et cetera*. But, and even though this procedure has led to good agreement for *FI* of Ref.^32^, more research on the applicability of the model should be carried out.

Also: The herein used approach is based on a simplified version of the theory of elasticity, which ignores stress concentrations (e.g. around knurls or defects in wood), Inglis’ potential energies, fatigue and crack propagations as described by Ref.^63^, which can lead to unexpected structural collapse if one relies solely on simple beam theory. The need to explore those ideas was suggested, as understanding their influence could be the key to understanding the relationship between structural failure and wind^42^. Fortunately, those relatively unexplored ideas were later applied to calculate critical wind speeds for failure in forest trees^64^. This could thus be an interesting starting point for research on the breaking prediction of palms and trees.

Moreover, the beam theory as used by some of the herein mentioned authors is aimed only at predicting conventional bending failure (axial compression stress that exceeds *MOR*), while low *t/R* ratios can lead to Brazier buckling or tangential cracking followed by longitudinal splitting in hollow stems^30^. The formulations were offered to predict the bending moment at which cracking failure would occur in hollow trees, based on *t/R*, *MOE* and tangential tensile *MOR*^54^. So, and for instance, if one took an oil palm and a coconut palm, both hollow and with an identical *t/R* and wind loading, the first would crack earlier than the second due to a lower tangential *MOR*. And as *t/R* decreased, failure modes would be bending failure, cracking and Brazier buckling respectively, for oil palm. Whereas in coconut palm, and depending on *t/R*, bending failure would occur earlier than the other two modes due to a comparatively higher tangential *MOR*/longitudinal *MOR* proportion. Which is also evidence why fixed *t/R* rules (e.g. 0.32 or 70% of the radius) and beam theory (e.g. pulling tests) cannot be applicable to palms. Hence, incorporating cracking and buckling predictions in the assessment of decayed and concentrically hollow palm stems, could also be an interesting lead.

Next, it was found that the Brazier calculations (based on *MOE*) agreed with the *BS* outputs (based on *MOR*) of the model for all heights along the stem and all wind speeds, when the stem was modeled as untapered, which is interesting. The relationships of varying *MOR* and *MOE* along the stem were based on the measured densities of Ref.^32^, so there seems to be a consistent mathematical relationship between the *MOR, MOE* and density values^32^. At first sight, densities of green palm wood could thus be an interesting future research subject. However, and on the other hand, it was reported that no correlation existed between density and mechanical properties in date palm wood^52^. Which would make future mechanistic modeling thus even more challenging.

Next: None of the herein investigated models and criteria fulfill the requirements (i.e. that models should account for cell wall expansion and sclerification as a result of height growth and age) stated by Ref.^60^. And they could therefore be precluded from being useful for palms, as the latter both grow and age.

Researchers also recorded longitudinal tensile stress on the surface of upright growing trunks, whereas compression stress was found at the bent area of leaning trunks in coconut palms due to growth stresses^65^. They also found compression stress in the outermost portion of the inner cylinder of the coconut stems, which they said was radically different from dicotyledonous and coniferous trees. So, this also questions the application of fixed *t/R* rules, pulling tests or wind load analysis combined with beam theory on palms, as those methods neglect growth stresses and their biomechanical importance. For instance, if the central cylinder were missing (e.g. due to butt rot caused by *Ganoderma zonatum*) then the lack of those inner and outer growth stresses and strains should be accounted for.

However, now we will suppose, and for the sake of the argument, that we approached the pitfall in which some of the aforementioned companies and researchers have seemingly already fallen. At a first glance, it would be appealing to suggest the following method: consider that commercial software packages for wind load and breakage predictions were successfully simulated^20^. And that special software packages were also suggested to accurately measure the vertical area of e.g. a palm crown, which would thus allow to perform a wind load estimation that would meet the standards of the commercial software packages investigated^20^. Suppose a wind speed-specific drag factor be introduced, such as proposed for Canary date palms by Ref.^17^ or the one found here for coconut palm. And that the formulations for the critical bending moments for tangential cracking from Ref.^54^ and the ones employed in this study for pure bending failure be incorporated, together with the wood properties as published for several palm species by e.g.^17,22,32,52,53,60–62^. Furthermore, values for peripheral material properties were obtained from the ring that corresponds to the outer third of the radius^32,52^. And take a non-linear bending stress distribution in the cross-section of the stem, which rises exponentially from the neutral fibre to the peripheral outer ring made of the most dense, stiffest and strongest tissues^21^. Then, a simplified assumption would be to calculate stresses taking into account only the outer third of the radius (i.e. *t/R* = 0.33), as if it were a hollow wind turbine tower and as has been done in the present study. And this, to simulate (in an extremely simplified manner) non-linear bending stress and peripheral material properties (note: it should absolutely be stressed here that this is not regarded as a validation of the VTA *t/R* = 0.32 rule, as the rationale for its use in the model differ from the rationale of Refs.^1,15^, while the inapplicability of the latter's claim has been amply evidenced in this study). In this way, theoretical safety factors could then be calculated and compared for bending versus cracking failures of the hollow palm stem, for varying wind speeds and several palm species. This would thus be similar to the widely-cited Statics Integrated Assessment (SIA) and Statics Integrated Methods (SIM) of Refs.^3,45^, but then for palms and slightly enhanced (as it adopts cracking failure, the varying material properties across and along the stem and a wind speed-specific drag factor). And as less advanced methods (e.g.^1,3,16^ have already been commercially marketed, a non-scholar could perhaps be tempted to commercialise this model in a software package or use it for their consultancy services too. However, this approach would still suffer from the same limitations as described in Refs.^19,20^ and in the present study. And it would still be theoretical, as the variables concerning the structural stability of hollow trees and palms may be too diverse to be assessed with current methods^19^. And the combination of small deviations in the real palms from e.g. the published values for *MOR* and *MOE* and theoretical drag factors (and hence predicted wind loads) could result in a global deviation that may invalidate the outcomes (the latter concerns all of the herein investigated methods too)^33^. Hence, and even though it is not the corresponding author's idea to wholly negate the usefulness of the herein investigated methods, it is crucial to point out that both their validation and predictive value are seemingly problematic.

A crucial rationale for presenting the utterly simplistic model in this paper was the following: supposedly complex models such as e.g.^3–5,45^ or the advanced 3DFE simulation of Ref.^32^ may obscure that fact that those models can be as tied to the same limitations as the simple model presented herein. And apparently complex equations (or e.g. a high number of citations of the related papers) may deviate the readers from the fact that factual empirical and scientific evidence could still be missing that would validate the models for real-life purposes. Hence, a simple model such as the herein presented one, may serve the purpose of pointing out the flaws and limitations of the seemingly more advanced models, while it even seems capable of simulating internationally-renown commercial software and methods^20^.

So, the time seems to be ripe now to go *beyond* the classical procedures as trusted upon by the arboricultural community so far (and discussed before).

Even in straight, thin and idealised cantilever beams, bending–torsion coupling deformations can arise due to the dissimilar bending stiffnesses when the two planes (horizontal/vertical) of the cross-section are of uneven dimensions (instead of a e.g. a perfectly circular or annular cross-section)^66^. Palm and tree stems are not always perfectly round due to dissimilar diameters in the horizontal versus the vertical plane (e.g. in cases of reaction wood, open cavities or uneven radial growth due to touching physical obstacles). Hence, simple beam theory (e.g. pulling tests) may thus not account for torsional (and, ultimately, catastrophic) behaviours, even in straight stems. Moreover, if improperly applied, simple beam theory may theoretically predict the strength and stiffness requirements of a structure to be satisfying, while unforeseen collapse may later occur because of the loss of stability (buckling), including intriguing phenomena such as non-linear geometric deformation and wrinkles^66^. Translated into arboricultural language: the tree or palm that had been assessed as “safe”, suddenly collapses unexpectedly. Therefore the need in this paper to show the arboricultural community that structural collapses, that have been studied for centuries in other fields such as mechanics and engineering, should not be ignored.

**Figure 2:**
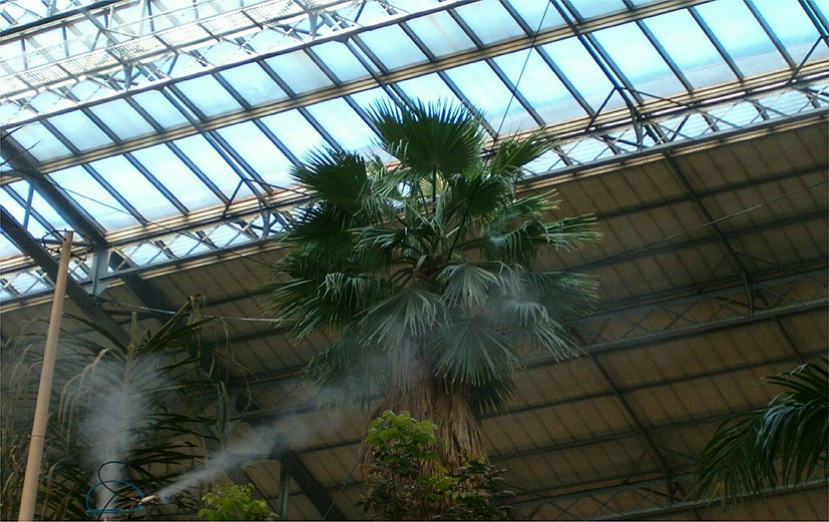
The slender Mexican fan palm anchored to the ceiling of the Atocha train station, Madrid. The cabling configuration was installed to minimise damage in case of post-buckling collapse. The other palms had collapsed before, even though there are no events of wind inside this giant greenhouse.

The risk of buckling of a Mexican Fan Palm (*Washingtonia robusta*), assessed by the corresponding author in 2003 in the Atocha train station (Madrid, Spain), gave birth to a proposal to assess the risk of Euler buckling while carrying out wind load estimations in order to optimise artificial supports (e.g. cabling of the palm to nearby structures)^28^. Prior to the assessment of the last standing palm, several other slender Mexican fan palms in that train station had already collapsed, even though the interior of this giant greenhouse is free of wind loads (Fig. 2). The photograph is a testimony to a rather neglected fact in commercial arboricultural methods: structural collapse in absence of wind loading and pure post-buckling failure. In this case it was hypothesized that these palms had initially become elastically unstable, by exceeding their critical stem height and weight. It was hypothesized, too, that this had been caused by their unlimited growth towards the glass ceiling searching for light, the absence of external loading stimuli such as wind (the lack of which would have made the palms not to invest in stiffer and denser wood) and optimum growing conditions (permanent moisture and warmth). The weight of the crown, small horizontal displacements, watering from the ceiling (i.e. fog to keep the atmosphere moist) and resulting gravity forces would then have further influenced the failure process, leading to final collapse. This example illustrates how plants can adapt to their environment and that biomechanical failure can be possible in total absence of wind loading. In large-wave Euler buckling, the column curves and deviates laterally to escape from compressive loads (such as e.g. self-weight) *before* axial stresses surpass axial *MOR*. The column becomes elastically unstable and buckles under its own weight. The critical weight divided by self-weight gives the safety factor and only when this safety factor is higher than unity can columns, or plants, bear additional loads such as wind, snow or ice. The critical buckling height or weight is a function of stem height, diameter, tapering, *MOE*, density of the wood and loading conditions^27^. The latter also showed that buckling safety can be overestimated if the stem is improperly assumed to be untapered, cylindrical, free of imperfections and isotropic^27^. They also offered an overview of why predictions of structural collapse may easily differ from real-life situations^27^. Moreover, the bifurcation point is the sudden jumping process of a beam from a straight-line to a bent shape, causing instability or buckling^66^. Pre-buckling analysis has proven to be rather straightforward for a simple pole, while the post-buckling process that describes the finite deformation of the structure (which may lead to its collapse after damage and faults accumulate to a certain value) requires a large set of numerical solutions^67^. Strong geometric nonlinearities and large displacements of the post-buckling behavior of a slender rod were studied, leading to a quantitative calculation of the post-buckling deflections of a hollow oil sucker rod^67^. Translated into the world of palm biomechanics, it means that: while pre-buckling of the stem would already be a daunting task due to the varying taper and *MOE*, predicting its post-buckling behaviour and final collapse (including structural faults such as e.g. cracks or pockets of rot) seems to be out of reach, as palm stems are not human-made structures. And yet, it seems reasonable not to ignore this type of structural behaviour in future palm risk assessments. It was acknowledged too that Brazier buckling played a crucial role in the local instability of plant stems^66^. And this was also a reason to include Brazier buckling of a hollow wind turbine tower to simulate breaking safeties of the coconut stem in the present paper.

Now, and as a second part of this “Discussion” section, the following reasonings elucidated from literature overview, visual observation and intellectual reasoning, are presented to postulate ideas that may serve to show the way towards a future unified theory on palm risk assessment.

First: The biomechanical structure of palms seems to have evolved towards highly-efficient energy dissipation and viscoelastic damping capacities under strong and dynamic wind loading. To achieve this, a triple-helical mesh of tough (high tensile strength) fibrovascular bundles is embedded in a soft parenchymatous foam, which both contribute to damping and energy dissipation^32,68^. The fibrovascular bundles run along the stem in a screw-like fashion and across the stem in a radial zigzag pattern (this also sets palm wood also apart from dicotyledonous wood, as in the latter the fibres are stiffly glued together and, most importantly, axially aligned). It was asserted that this screw-like pattern can hold the bending stem together under high wind loading as it lends the stem a higher stiffness and strength when the fibrovascular bundle orientations varied between 0° and 9°^32^. This pattern was also suggested to minimise longitudinal splitting and thus enhance the mechanical efficiency of the stem^32^. This structure was an inspiration for spirally-laminated hollow veneer-based composite poles^32^. Also high microfibril angles across the fibre cap would result in a high extensibility of the stiffening tissue, which would enable palms to cope with considerable deformations under wind loads in Mexican fan palms^69^. Large deformations in bending and torsion under wind loads of the petioles were said to combine efficiently with water and nutrition conduction, due to the optimized connection of their vascular bundles to the leaf traces^68^, which allows to suppose that also the crown is optimized regarding damping and energy dissipation brought about by dynamic winds. And the contribution of both parenchymatous and vascular tissue of palms to energy dissipation, dynamic response and flexibility, and thereby improving impact resistance, was described too^70^.

**Figure 3:**
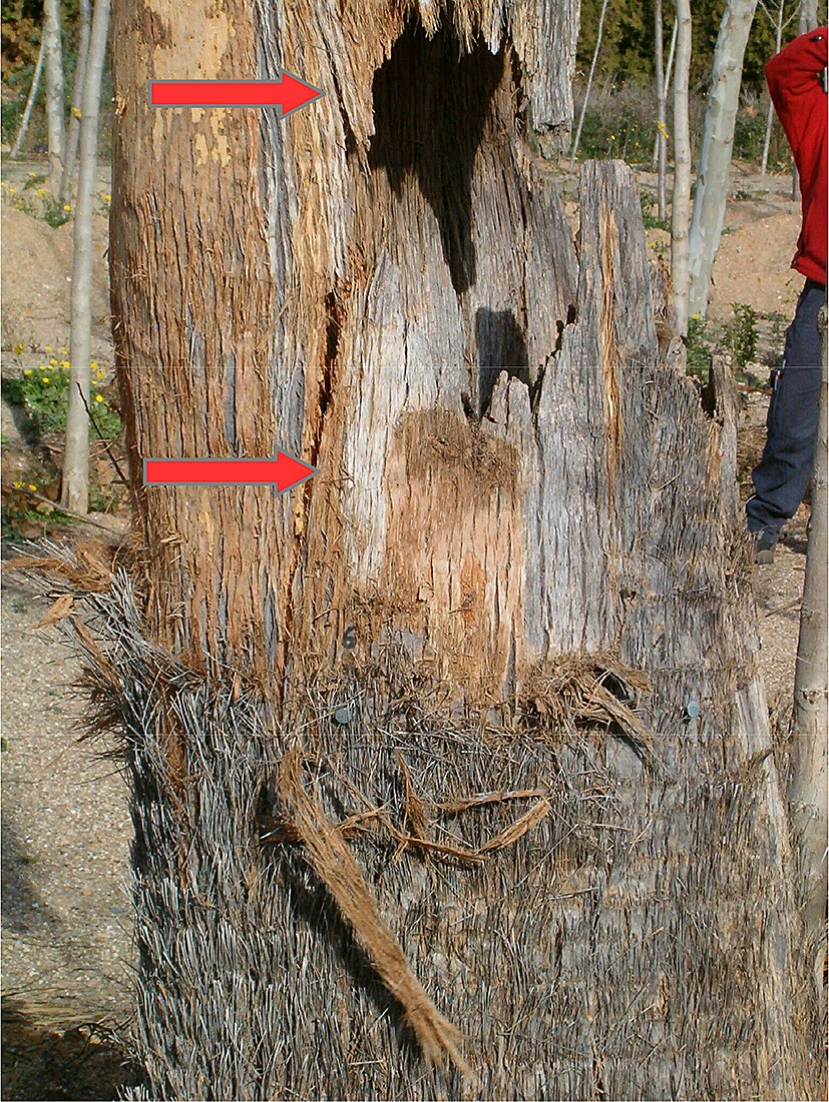
When a decayed desert fan palm stem was statically pulled, collapse was initiated by splitting of the hollow stem. Cracks first appeared above and below the open cavity and initiated at its borders (red arrows) and total collapse only ensued after large longitudinal splitting and delamination.

**Figure 4:**
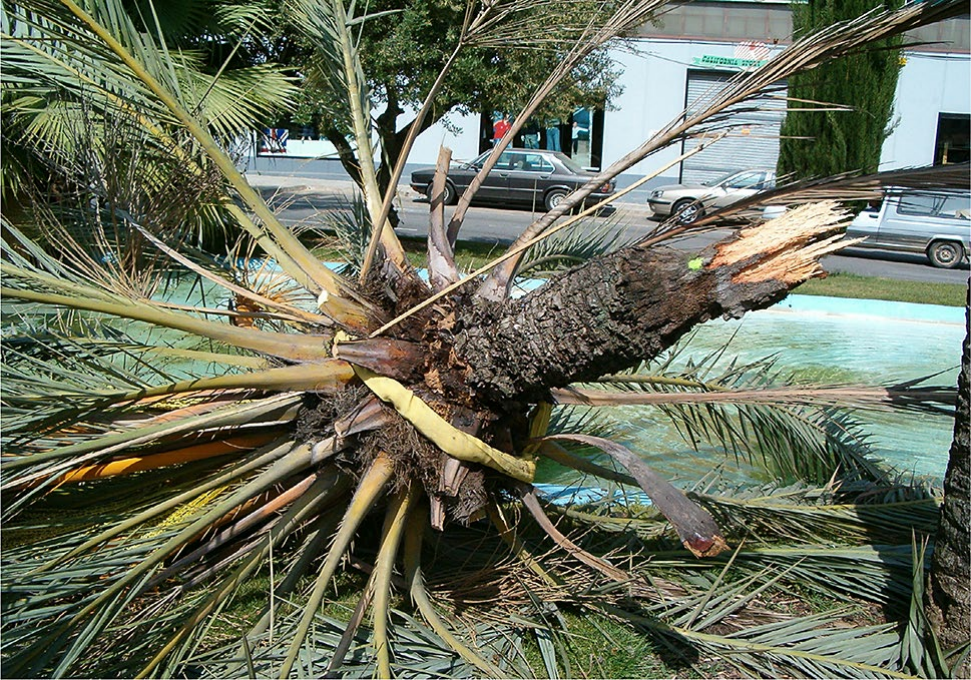
When a decayed date palm stem was statically pulled, splitting was initiated at the open cavity and total collapse ensued by delamination.

Second: Palm wood is highly sensitive to shear, delamination and splitting in comparison with dicotyledons. For instance, when samples were taken by Ref.^17^ to perform longitudinal compression and tension tests, then this irregular structure of the palm tissues unwillingly led to longitudinal fractures, sliding and shear in the samples, and thus seriously limiting experimental data on axial *MOR*. And thick disks of coconut wood were manually torn apart, while the delamination followed the helical pattern of the fibrovascular bundles that tangentially deviated across the disc diameter^32^. Hence, it is thus not unreasonable to suppose that this sensitivity to delamination and shear may lead to the stem's structural collapse, especially when this helical path of bundles is interrupted by a mechanical defect (e.g. pockets of rot, irregular decay, cracks or tunneling by Red Palm Weevil). This reasoning aligns with another researcher's too, who likened coconut and oil palm stems to a composite material made of a matrix and reinforced elements and found that shear and tension perpendicular to grain greatly govern the bending behaviour and structural stability of the stem^52^. The aforementioned “spirally-laminated hollow veneer-based composite poles” suggested by Ref.^32^ may be very stiff and strong when undamaged (i.e. if this helical pathway of fibrovascular bundles is not interrupted by a mechanical defect and thus a completely defect-free beam). But, an interruption along this path may trigger delamination and splitting along the “veneer”. Crack propagation and splitting could thus follow the helical path of the fibrovascular bundles. And predictions based solely on simple beam theory and axial stress and strain would then be less than acceptably reliable. Observations and experiments that seem to support this hypothesis are e.g. the aforementioned Canary date palm that crushed a man in Barcelona, as a small inner crack was said to have triggered the sturdy stem’s collapse with a breeze of only 38.2 km/h^10^. Also pulling test experiments carried out in 2004 by the corresponding author showed that the mechanically damaged palm stems under artificial loading started splitting first, leading to full collapse afterwards^42^. Those experiments (partially published in 2005^42^) had been kindly supported by Josep Selga S.L., the City Councils of Terrassa and Mataró and the Asociación Española de Arboricultura, while the instrumentation had been kindly provided too (Picus tomograph: L. Göcke Argus Electronics; Pulling tests: Brudi and Partner Tree consult and Dr. Ing. L. Wessolly; Resistograph F300, IML: the City Council Terrassa). The aim was to assess whether the pulling tests of Refs.^3,45^ could be adapted to palms or not and if experimental data for *MOE* could be obtained from standing palms. Acoustic tomography (Picus tomograph) and microdrilling (Resistograph F300) had also been carried out on several damaged palms, but had not facilitated any reliable breakage prediction either (unpublished results). An example is shown in Fig. 3 where a desert fan palm (*Washingtonia filifera*) collapsed under a static pull, after slanted longitudinal splitting and delamination was initiated at the border of the open cavity (upwards and downwards)^42^. Also Fig. 4 shows how delamination (triggered at the height of the open cavity under a static pull) led to total collapse of a date palm stem. No primary axial compression failure was observed macroscopically^42^. And a hollow date palm exhibited extremely high shear values in comparison with axial deformation at the height of a large, open cavity (Fig. 1)^42^. Moreover, it is not unreasonable to suppose that if strong, cyclic and repetitive dynamic wind loading had beaten these three palms (instead of a static pull), the risk of structural failure could have been heightened by progressive fatigue of the wood around the structural defects (and thus earlier crack formations/propagations and at lower loads than with the static pull).

Third: Highly deformable and soft but elastic materials can exhibit types of structural deformation under mechanical loads that are unlike those commonly observed in elastic structures that behave linearly^71^. Kinking at the inner side of soft, elastic cylinders was observed after the cylinders had become elastically unstable due to Euler buckling^71^. The extreme localization of curvature at the compressed *inner* (not outer) side exceeded a critical value leading to a sharp fold. When the cylinder was kept under a bending load for several minutes, irreversible defects appeared at the location of the inner kink which, in subsequent loading cycles, progressively lowered the cylinder's structural stability under the same amount of load^71^. Translated into palm stems, and assuming they are highly deformable, soft and elastic, this means that inner kinks and defects could appear and lead to structural collapse due to fatigue and cyclic loading beyond the critical curvature. Brazier buckling was also observed in soft, elastic and hollow cylinders and the occurrence of either kinking and/or ovalization was found to be dependent on the ratio between the diameter and the wall thickness^71^. When one envisages palm stems as has been done in the present paper (a viscoelastic cylinder), then the kinking and ovalization of the cylinder (here: the palm stem), after becoming elastically unstable, could thus lead to abrupt structural collapse while not obeying simple beam theory. Calculation of the critical curvature at which buckling sets in was said to be rather straightforward, but the posterior evolution of the kink or defect would need detailed non-linear theory^71^. Hence, the modeling of elastic pre-buckling (i.e. prior to these aforementioned structural failures) seems to be more within reach for palm stems than post-buckling collapses. No experiments have been performed on palms yet to either confirm or refute these extrapolated suggestions, but the latter are possibly worth considering in future research or risk assessments.

Fourth: Developing a mechanical model seems currently out of reach as strength and stiffness (and thus damping) seem to evolve over time in the palm stem as a function of the location of the vascular bundles within the trunk, age (and ensueing additional cell wall layers and (secondary) growth within the trunk) and growth conditions^52^. Also the lack of a statistical correlation between *MOR* and *MOE* and wood density in date palms is, inexplicably, contrary to other investigated palm species, which also obstructs the path towards reliable mechanistic modelization^52^.

Fifth: It was stated that “Reliable prediction of delamination growth is still proving to be problematic” in human-made wood products, whereas simply localising starting points for delamination would possibly be more within reach^72^. From which it can thus be inferred, that reliable predictions of delamination-triggered collapses of Nature-made palm and tree trunks seem currently to be out of reach. But that would still be no reason to neglect this type of structural failure).

Sixth: The existence of silica in palms was mentioned by Ref.^52^ (p. 158) and studied by e.g.^73,74^*.* Researchers concluded from a literature review that the mechanical properties of palms could be enchanced by silica^73^. And the role of silica in plants was described as: “Biomineralization is a naturally occurring process by which living organisms form skeletons from inorganic minerals such as silica and calcium”^75^. The latter also found flexural rigidity in rice plant leaves to increase with increasing silica content. It has been suggested by practitioners and arborists in Spain that silica and biomineralization would make the palm stem stiffer and stronger around structurally defective areas, as an alleged reaction to strength loss percieved by the palm itself (i.e*.* a substitute for compensation or thigmomorphogenesis as studied in dicotyledons), but no scientific findings were found that would support their suggestion.

Seventh: Local mechanical performance (i.e. damping and the diminution of stress discontinuities) of a Mexican fan palm stem could be controlled by the plant itself up to a certain point by adaptation^69^. Which would further complicate the mechanical modeling of structural stability versus (wind) loads.

Eighth: The cracking formulation of Ref.^54^ should unfortunately be precluded from being useful in hollow palms, as their formulation assumes that the fibres are aligned along the tree axis, while palms present a mesh of triple-helical fibrovascular bundles in a screw-like pattern along and across the palm stem.

Ninth: Based on visual observation, young and still flexible and soft Mexican fan and windmill (*Trachycarpus fortunei*) palm stems seem to exhibit a viscoelastic behaviour when manually pushed and pulled. Their moving out-of-phase with the pulls can be felt by hand and feels like a structure made of foam, but with a certain resilience. Their behaviour resembles neither that of a steel spring nor that of foam or a stiff and non-deformable beam. And this in contrast with e.g. flexible dicotyledonous saplings and tree branches that almost behave like springs or lashes when laterally loaded and released by hand. Also visual observation of the damped manner in which older, taller and stiffer Mexican fan, date and windmill (*Trachycarpus fortunei*) palm stems move out-of-phase in strong winds seems to confirm this. And in several palm species the woven mesh of leaf sheath palm fibres attached to the stem also exhibits a damping and viscoelastic behaviour when manually manipulated. In windmill palm for instance, the stem is wrapped in a burlap-like mesh of brown and coarse leaf sheath fibre, clasped around the trunk. Manual manipulation of that mesh suggests that friction among the fibres could contribute to damping of leaf and stem movements. A review of published findings on damping and energy dissipation in palms seems to confirm these visual obervations too (see^32,68–70^). A viscoelastic structure exhibits a non-linear response to the strain rate, in which cyclic stress is out-of-phase with strain, as some of the stored energy is recovered upon removal of the load, while the remaining energy is dissipated as heat. The modulus is represented by a complex quantity: on the one hand the stiffness is defined by elastic behaviour and, on the other hand, the energy dissipative ability of the material is defined by the material’s viscous behaviour. Hence, one could thus hypothesise that the palm stem could be neither an elastic nor a viscous structure, but a combination of both.

So now, the aforementioned observations lead us to the following:

The herein postulated model envisages the palm stem as a viscoelastic and hollow cylinder prone to Euler and Brazier buckling and ovalization and kinking. This hypothetical model could graphically be imagined as a hollow foam pool noodle with a triple-helicoidal embedded mesh of tough (a high tensile strength) fibre bundles. Both the foam of the pool noodle and the mesh of fibres contribute to the damping while the latter also adds flexural stiffness under bending. The cylinder exhibites a non-linear response to the strain rate, in which cyclic stress is out-of-phase with strain, which makes the whole structure viscoelastic. This envisaging was the main reason why Eq. (10) for Brazier buckling, with a constant *t/R* for all wind speeds, was experimentally applied to simulate *FI* of the cocostem of Ref.^32^.

However, it would also be prone to delamination, splitting and shear as the bundles are glued together with “foam” along their screw-like path. The momentum the cylinder should withstand should be a result of dynamic wind loads, mass and inertia that cause a non-linear deformation and pronounced curvature of the cylinder (non-linear due to the varying material properties along the (tapered) stem and structural damping). Strains in the stem would then not be linearly proportional to the load, by which Hooke's law (*ut tensio sic vis*) would not be not applicable. And stress would rise non-linearly along the stem radius from the core to the periphery. Progressive fatigue of the wood, or at structural defects (e.g. crack initiations and progressive propagation due to repeated dynamic wind loading), should be taken into account. This model would now possibly align quite well with the scientific findings cited in this paper.

Nevertheless, a simple mind experiment can reveal the additional baffling challenges found in real palms: imagine a date palm trunk that has been severely tunneled by Red Palm Weevil and/or pockets of rot: the structure resembles a piece of Gruyère cheese and allows remaining bundles of sound strands to be torn off by hand, as the stiff vascular bundles are just lightly glued together by means of a foamy parenchymatous tissue. The remaining bundles and volumes of sound wood, bordering the void and decayed spaces, could then resemble irregularly shaped columns. Now, imagine the loading of this disk of “Gruyère cheese” due to a bending moment: an infinite variety of kinds of structural failure would take place within the remaining “columns”: buckling, sliding, shear, sideways kinking of the fibres, torsion, crack propagations along a triple-helical path, stress concentrations, *et cetera*. And as the smart reader will surely agree to, this three-dimensional failure process is totally impossible to depict, or assess, by means of drilling, tomography or simple beam theory. Doubtful readers can have a look at the Figs. 10 and 11 in Ref.^17^ and imagine that the wood blocks in those figures were the remaining “columns” of our imagined trunk. And, as it can be seen in those figures, the blocks structurally failed due to shear, even under pure axial compression and tension^17^. And now let us add the following: looping movements of a tall palm in winds has already been recorded^18^. These looping and circular motions of the stem, inevitably, cause a rotative loading of the cross-section of that same stem. This rotative motion thus causes compression stress (and tension stress on the opposite side) at the periphery and in a circular motion, Real wind loading of palms is thus very dissimilar to the unidirectional loadings (assumed or performed) by e.g.^1,3,16–18,32,36^. And let us add too, that shearing behaviours can be caused due to structural defects (e.g. see Fig. 1), and couple this with the rotative motion and possible progressive fatigue processes in the root system and stem. Now the abovementioned reasonings leave us with a mind-boggling panorama of infinite variables, which seemingly precludes all herein investigated methods from being reliable. However, and from a constructive point of view, these postulated ideas are possibly the best starting point for the development of a future risk assessment method. And the herein offered observations can be used by arboricultural professionals to enhance their tree and palm risk assessment consultancy reports.

This is only a partial theory, which need not cancel out others *per sé*, but may overlap others so as to reach a more acceptable degree of predictive accuracy. This may be a step toward a more complete, fully-unified and more reliable theory that would enable us to make predictions that agree with observations to an acceptable degree of accuracy. Constructing a complete theory from scratch looks excruciatingly difficult now, so perhaps the way forward would be to *overlap* existing partial theories. Partial theories describe a limited variety of events while leaving others aside. Current partial theories in arboriculture do not seem to be valid on their own^19,20,33^. Examples are theories that neglect common mechanical behaviours of the wooden body^33^, simple beam theory and dubious t/R criteria for palm risk assessments. Or predictions of uprooting and breakage that are based on a static wind load analysis, if the latter does not take into account the influence of slenderness, dynamics, mass and inertia in slender and top-loaded (due to e.g. a lion-tailed crown or heavy crop) palms and trees^20^. A complete theory would thus contain a number of parameters which values, in real-life, cannot be predicted yet and such values may have to be chosen to fit in through experiment. A very appealing goal would be now to overcome this mind-boggling and infinite combination of behaviours and (structural and material) properties, and distill it all into one simple and generic law/model, as was elegantly done for buckling by Ref.^24^.

Researchers have taken sound stems as a starting point (e.g.^17,18,32^. But, perhaps structurally-damaged trunks should be the place from where to start, as the latter are generally the aim and goal of risk assessments. Future methods could thus perhaps focus on deformations of the stem under circular (wind or artificial) loading, while three-dimensional mechanical behaviours and failures can reasonably be expected within a damaged stem. And also three-dimensional material properties should be taken into account: i.e*. MOR* and *MOE* in all anatomical directions. But, as taking those values from published tables would not be feasible (due to the high variability of those properties), different methods from the ones used by e.g.^1,3,5,16–18,32,36^ should perhaps be devised. For instance, a preliminary investigation was carried out on forced vibrations, and resulting resonance frequency values, for a Mexican fan palm, in the light of the identification of trunk decay and its level of severity^76^. And this could perhaps open up new leads for research. Vibration analysis could monitor repetitive motion signals, to detect abnormal vibration patterns and levels, which could allow the assessment of the overall structural condition of the trunk. But then one would still be left wondering whether that approach would reliably assess e.g. the risk of delamination and crack propagation, or ovalization and kinking.

Nevertheless, it is now clear that if we stay within the limits of the theories that are the basis of methods such as e.g. the tree-statics of^3^, *t/R* rules used by Ref.^1,15^ or the ill-fated pulling tests as reported by Ref.^77^, then our mind will possibly not be able to devise the path of evolution.

## Conclusion

It appears that in the current study, and for the first time, the tenability of influential claims has been analysed regarding palm biomechanics in the context of risk assessment (breaking and uprooting). Theoretical wind loading and breaking strength of a coconut palm had been computer-generated with a 3D Finite Element software^32^. The latter's results were simulated herein with a simple model on a spreadsheet, by using *MOR* (classic beam theory) and *MOE* (Brazier buckling) and a hollow, tapered cylinder and applying a wind speed-dependent drag factor. However, elementary scholars and students can easily follow the simple formulations, and that was the intention too: the model was devised to support the herein offered analysis of the scientific veracity of some influential claims and commercial tree and palm risk assessment methods that have dominated the arboricultural industry and press in the last 20 years. But most importantly, and on the remains of the previously questioned or refuted claims, fresh observations based on review and reasoning have been presented to construct new ideas, which have been postulated herein as a possible path towards a new theory on palm risk assessment. The postulated model envisages the palm stem as a viscoelastic and hollow cylinder that is not only prone to buckling, ovalization and kinking, but also fatigue, shear, splitting and crack propagation. This envisaging was the main reason why a simple Brazier buckling formulation was experimentally, and successfully, applied herein to simulate the breaking risk of a coconut palm stem.

A review of available publications also suggests that the strongest claims on palm risk assessment methods could be found in non-scientific grey literature, such as magazines for arborists, websites of arboricultural firms and lay press. One could thus wonder why the latter have had such an impact, been so influential and marketed at such a grand scale. Scientific papers from peer-reviewed journals, on the other hand, did not offer the supposed solutions and impacting claims. It is interesting to note, that the publications that offer dubious but influential claims would not have been detected, had the review been conducted in a traditional academic manner (i.e. searching only in academic databases). The field related to biomechanical tree and palm risk assessment seems thus especially wanting in terms of independent scientific research.

Several claims currently seem to have the quality of unsupported suggestions, against which contrary evidence can easily be found, or are suggestive of irresponsible practice. So, it would not be unreasonable to suppose that unforeseen collapses and accidents could thus be a result. For instance, it was reported how two date palms were unexpectedly torn down in Spain while carrying out a risk assessment with an undetermined kind of pulling test^77^. Fortunately, a bystander caught the unfortunate event on video and it graphically shows how that pulling test causes the palm to collapse and (unintentionally) destroy property^77^.

This study should also open eyes to possible consequences (e.g. legal consequences in cases of loss of life or property) that could sprout from marketing criteria and methods which foundations seem difficult to reconcile with sound scientific practice, observation and reasoning. Evidence of Irresponsible Research Practices (*IRP*) and flawed or questionable claims have been laid bare here and in^19,20^. Simultaneously, a debate is emerging on whether *IRP* and Fabrication, Falsification and Plagiarism (*FFP*) should be taken more seriously if it leads to grossly negligent or potentially harmful practices or products^78^. The latter also said that societal or ecological consequences of *IRP* would be relevant in setting the boundaries of its criminalisation. This is also why, among other reasons, a culture of unbiasedness, reproducibility, rigour and transparency should be promoted in research.

Another unsettling finding of the review was that a wide array of papers on palms was found in journals that have been listed as predatory by Ref.^79^. The shady side of predatory journals is that lightly-reviewed articles or floppy editing can promote influential claims that are based on *IRP* or *FFP*. For instance, the low scientific credibility of a predatory journal was evidenced by Ref.^80^ who managed to publish a bogus paper, just to prove that peer-review was apparently non-existent.

It has been shown that palms can withstand considerable deformations under wind loads, due to the biomechanical peculiarities and orientation pattern of the fibre caps of the single vascular bundles, embedded in the soft parenchymatous tissue^69^. And yet, a breeze of maximum 38.2 km/h and a small structural defect apparently sufficed to break a sturdy Canary date palm and kill a person^10^. And a rind-core design in plants (i.e. a rind of lignified tissues surrounding an incompressible core of parenchymatous tissues that could change in shape but no in volume) would theoretically reduce the probability of Euler and Brazier buckling^81^. And yet, the tall Mexican fan palms of the Atocha station in Madrid had buckled and collapsed in total absence of wind. This suggests that the real reasons behind, and proper assessment of, the uprooting or breaking of trees and palms still remain elusive.

Lastly, the present paper is merely the result of a review combined with intellectual and theoretical exercising, as the corresponding author has had no financial support to perform the experiments needed to either confirm or refute some of the herein postulated ideas. Nevertheless, it is hoped that this study may offer ideas for future experiments and also motivate the arboricultural community in the search for more reliable tree and palm risk assessment methods through independent and unbiased research.

## Methods

Research was carried out by simulating the results of the 3D Finite Element Analysis of Ref.^32^ and a review of publications and commercial methodologies was conducted. The outcomes of the model highlight the observations drawn from the review.

### Wind load analysis

The mathematical structure was published in Ref.^20,33^ and the necessary parameters were taken from Ref.^32^. The procedure of adapting the wind turbine model of Ref.^20^ was intentionally maintained simple. Firstly, the wind profile was calculated as:

1 $$v_{z} = v_{g} \left( {\frac{{h_{z} }}{{h_{g} }}} \right)^{\alpha } G$$

where, *v*_*z*_ is the wind speed at a certain height above ground level (m/s), in this case ranging from 10 to 60 m/s, taken from Ref.^32^; *v*_*g*_ is the maximum wind speed expected, not influenced by the roughness of the surrounding terrain (m/s); *h*_*z*_ is the height above ground level at which *v*_*z*_ is reached (m); *h*_*g*_ is the height above ground level at which *v*_*g*_ is reached (280 m); *α* is the surface friction coefficient (0.16); *G* is a gust factor (1.22).

The wind load formula was calculated as:

2 $$F = \left( {0.5{\text{C}}_{d} \uprho A{\text{v}}^{{2}} } \right)\frac{9.80665}{{1000}}$$

where, *F* is the force of the wind in the crown (kN); *C*_*d*_ is the drag coefficient stands for the flexibility that the palm or tree employs in order to diminish the force of the wind and is dimensionless; ρ is the air density at a certain altitude and temperature (kg/m^3^); *A* is the vertical area of the palm (crown and stem) (m^2^); v is the wind speed (m/s^1^).

The vertical area of the crown (*A*) was 3 m^2^, the stem area 5.95 m^2^ (taking the basal and top diameters and the length of the stem), *P* was 25 m and air density was 1.20 kg/m^3^ according to Ref.^32^.

The lever creates a bending moment (*M* in kNm) calculated as:

3 $$M = PF$$

where, *M* is the bending moment (kNm); *P* is the lever (25 m); *F* is the force of the wind in the crown (kN).

### Wind speed-specific drag factor

The model simulated the bending moments of Ref.^32^ as follows:

First, drag factors were introduced in the model of Ref.^20^, to agree with the bending moments for all corresponding wind speeds of Ref.^32^. A wind speed-specific drag coefficient represents the streamlining, and resulting decrease in frontal area, of the crown and stem as the wind speed increases. The relationship found was:

4 $$y = 0.11187391 + \frac{0.63561235 - 0.11187391}{{1 + \left( {\frac{x}{30.416375}} \right)2.5433076}}$$

where: y is the drag factor, dimensionless; x is the wind speed at 10 m height, in m/s.

The coefficient of determination was *R*^2^ = 0.9997. This automatically calculated wind-speed-specific drag factor was then copied into the wind load analysis model of Ref.^20^.

### Breaking strength

Gonzalez^32^ calculated *FI* (dimensionless) with non-static linear analysis (due to the large deformations of the coconut stem) and by taking into account three-dimensional stresses (longitudinal, radial and tangential) and Poisson's ratios. An *FI* of greater than 1 means that the stem fibres hypothetically suffer failure without necessarily leading to the collapse of the stem. The model simulates *FI* of Ref.^32^ by simply modelling the stem as the shell of a hollow wind turbine tower. To fit in the wind turbine model's simplicity, three-dimensional stresses (longitudinal, radial and tangential) and Poisson's ratios were translated into pure axial compression stress, with the following procedure:

The increasing stresses, caused by increasing non-linear deformations of the stem under an increasing load, were calculated by varying its shell thickness. That is, a certain wall thickness (*t*) over the radius of the stem (*R*) gives a *t/R* ratio and a corresponding cross-section modulus (*W* in mm^3^). If the shell gets thinner, longitudinal peripheral bending stress rises and vice versa. At all heights of the hollow tower, a certain *W* and a certain compression strength or modulus of rupture (*MOR* in kN/mm^2^) are needed to resist *M* in simple bending.

The cross-section modulus of the stem *W* was calculated as follows:

5 $$W = \frac{{\left( {\uppi d_{net}^{3} } \right)}}{32} * 1000$$

where, *W* is cross-section modulus (mm^3^); *d*_*net*_ is the net diameter (cm).

Diameter of the stem at a given height was calculated according to Ref.^32^ (p. 43) and thus modeled as tapered.

Then, compression stress (σ in kN/mm^2^) was calculated as:

6 $$\upsigma = \frac{M * 100}{{W_{ring} }}$$

Finally, breaking safety (*BS* in %) was calculated as:

7 $$BS = \left( {\frac{MOR}{\upsigma }} \right) * 100$$

Peripheral *MOR* values varied along the stem height according to Ref.^32^ (p. 83). A *BS* of less than 100%, means that the outer stem fibres hypothetically suffer failure under pure axial compression stress.

Failure Index (*FI*) was then calculated as:

8 $$FI = \frac{100}{{BS}}$$

Necessary *t/R* ratios at all wind speeds had to be found beforehand, so that bending moments at varying wind speeds would match *FI* of Ref.^32^. The correlation of wind speed-dependent t/R ratios for wind speeds equal or greater than 20 m/s was found to be:

9 $$y = 0.20706989 + \frac{1120164.9 - 0.20706989}{{1 + \left( {\frac{x}{0.25220961}} \right)3.346368}}$$

where: y is the t/R ratio, dimensionless; x is the wind speed, in m/s.

The coefficient of determination was R^2^ = 0.9979.

Next, a linear correlation was established for wind speeds from 10 to 19.99 m/s by taking as a starting point the necessary t/R for agreement with *FI* at 10 m/s. This linear correlation is different from the exponential one (the latter for wind speeds above 20 m/s), as stem deformation (and stresses) are very low between 10 and 19.99 m/s due to the small curvature of the stem.

The *t/R* ratio allows to calculate *W*_*ring*_ of the concentric outer ring of the hollow tube by discounting *W*_*hollow*_ of the hollow area from *W*. Note that this just a mathematical adaptation (not a real *t/R*) to translate stem deformation and resulting three-dimensional stresses into theoretical longitudinal compression stress to fit into the simple wind turbine model. This simplicity also allows for easier comprehension of the remarks offered hereafter.

Thus, Ref.^32^ calculated *FI* due to large displacements of the cocostem subjected to strong steady winds and visible to the naked eye, with non-linear geometry analysis. In the present model, these non-linear changes in stresses caused by the large deformations were represented by changes in axial compression stress due to bending moments affecting the shell of a wind turbine tower. This is the reverse-engineering of the proposal published by Ref.^22^ where the latter calculated the buckling resistance of man-made shells (such as wind turbine towers), taking the palm stem anatomy and material properties as a starting point.

### Brazier buckling

The wind turbine model also simulates *FI* by modelling the stem as a round shell subject to Brazier buckling, with the equation given by Ref.^29^ (p. 293). The critical stress for Brazier buckling was calculated as:

10 $$\upsigma_{critbrazier} = \left( \frac{1}{4} \right) * \left( {\frac{MOE}{{10}}} \right) * \left( {\frac{t}{{R_{cav} }}} \right)$$

where, σ_critbrazier_ is the critical stress that causes Brazier buckling of the shell, kN/cm^2^; *MOE* is the modulus of elasticity, in kN/cm^2^; *t* is the thickness of the shell which remains constant for all wind speeds, in cm; *R*_*cav*_ is the radius of the cavity, in cm.

Longitudinal *MOE* values varied along the stem height according to Ref.^32^ (p. 79). The necessary thickness of the shell, for agreement with *BS*, was introduced (1,632535 cm). The radius of the stem at 13.1 m height was 10.45 cm.

The safety factor against Brazier buckling is then:

$$Safety_{brazierbuckling} = \frac{{\sigma_{critbrazier} }}{{\sigma_{compression} }} * 100$$

where, σ_*compression*_ is the longitudinal compression stress taken from Eq. (6), in kN/cm^2^; *Safety*_*brazierbuckling*_ is the safety factor against Brazier buckling, in %.

## Literature review

The review of publications related to palm risk assessment was conducted as follows: searching on Google (incognito mode) was chosen, as this enabled to detect highly-influential authors and claims in grey literature too (e.g. articles in magazines, websites and newspapers) that would not have been detected in databases that are commonly used for academic purposes and reviews (e.g. Scopus or Pubmed). Search terms evolved around the following: ‘biomecanica palmera datilera’, ‘biomechanics coconut palm’, ‘drilling decay palm’ and ‘visual tree assessment palm’ (in English and Spanish). Then, the process was continued by manually selecting other publications that were cited in the publications retrieved during the first search. The cycle was repeated until sufficient evidence was found to support the analyses. Publications were sought for that offered commercial tools, alleged solutions for the risk assessment of palms or have been influential in e.g. the arboricultural industry, the mass media or court cases. Some of the reviewed publications had already been retrieved for Refs.^19,20^. Unfortunately, some publications are available to members only and could thus not be scrutinised (e.g. *PALMS* of the International Palm Society).

## Data Availability

The datasets generated during and/or analysed during the current study are available in the Open Science Frame repository, https://osf.io/gehdu/?view_only=99cfd4418a08483583c4b86a372582f5.
