# Author Correction: The quest for a unified theory on biomechanical palm risk assessment through theoretical analysis and observation

**DOI:** 10.1038/s41598-021-02407-8

**Published:** 2021-11-23

**Authors:** Peter Sterken

**Affiliations:** Unaffiliated, Plasencia, Spain

Correction to: *Scientific Reports* https://doi.org/10.1038/s41598-021-01679-4, published online 11 November 2021

The Data Availability section in the original version of this Article was omitted.

It now appears as below:

“The datasets generated during and/or analysed during the current study are available in the Open Science Frame repository, https://osf.io/gehdu/?view_only=99cfd4418a08483583c4b86a372582f5.”

The original Article has been corrected.

